# Patient perspectives on health care models in cardiac surgery: a qualitative evaluation

**DOI:** 10.1186/s12913-024-11791-6

**Published:** 2024-10-30

**Authors:** Mona Elisabeth Schmid, Jannik Stumm, Sina Stock, Evaldas Girdauskas

**Affiliations:** Department of Cardiothoracic Surgery, University Hospital Augsburg, Stenglinstr. 2, 86156 Augsburg, Germany

**Keywords:** ERAS, Cardiac surgery, Patient evaluation, Heart valve surgery, Interprofessional care, Perioperative care, Patient empowerment

## Abstract

**Background:**

The implementation of ERAS represents a promising solution to improve treatment efficiency and facilitate patient involvement. This innovative care model aims to optimize recovery processes following surgeries by adopting a holistic, interprofessional approach. At our hospital, ERAS was implemented in minimally invasive heart valve surgery, offering two distinct ERAS models. Additionally, there is also the standard of care without ERAS. The objective of the study is to gain insight into patient satisfaction and perceived differences across these various care models.

**Methods:**

Patients were interviewed using semi-structured interviews approximately two to three months after undergoing surgery. The data were analysed using qualitative content analysis in accordance with the methodology proposed by Kuckartz. Four main categories were established: Preoperative care, postoperative care and communication, patient participation and involvement, and rehabilitation and post-clinical course.

**Results:**

Comprehensive preoperative education and seamless communication throughout the perioperative care journey were identified as fundamental to patient satisfaction and optimal care processes. Patients in the ERAS + model reported higher overall satisfaction with their care compared to patients in the standard of care and ERAS groups.

**Conclusion:**

Preoperative education establishes the foundation for patients’ subsequent behaviours and expectations regarding their treatment. Physical activity, nutrition, and mental health are significant aspects. The active involvement and participation of patients and their families in the treatment process facilitated superior postoperative care, intensive physiotherapy, mental support, and faster recovery. A functional flow of information throughout the entire care process is vital. Moreover, having a dedicated point of contact had a beneficial impact on patients´ well-being. The integration of innovative ERAS concepts, which encompass interprofessional preoperative patient education and psychosomatic support, represents a promising approach from a patient perspective, offering benefits to a broad spectrum of cardiac surgical patients.

**Supplementary Information:**

The online version contains supplementary material available at 10.1186/s12913-024-11791-6.

## Background

The German healthcare system is facing significant challenges due to a shortage of skilled personnel in hospitals [1]. For instance, in 2022, Germany lacked up to 50,000 nurses in intensive care units, which was negatively impacting the quality of patient care [2]. One proposed solution is the implementation of innovative, evidence-based care models with the objective of enhancing treatment efficiency and patient involvement. An illustrative example is the Enhanced Recovery After Surgery (ERAS) model, which is designed to improve patient recovery and optimize physiological function [3, 4].

ERAS aims to optimize the patient recovery processes by minimizing stress and maintaining physiological functions to shorten the postoperative recovery phase [5, 6]. In comparison to the conventional approach to surgical care, the ERAS model adopts a multidisciplinary approach involving surgery, anesthesia, intensive care, nursing, physiotherapy, and nutrition [7], focusing on patient-centered care and active participation.

Furthermore, the implementation of ERAS models has the potential to result in cost savings, as evidenced by reduction in hospital stays by up to 50% of their length and a decrease in postoperative complications, such as delirium. This ultimately enables patients to regain independence and return to work at an earlier stage [8].

In cardiac surgery, ERAS represents a relatively novel yet promising approach, particularly given the substantial prevalence of heart disease as a primary cause of hospital admissions [9]. ERAS models have been successfully implemented in a number of surgical disciplines, but is a relatively recent development in the field of cardiac surgery, with the first guidelines published in 2019 [10]. In January 2021, we started to implement ERAS in cardiac surgery (minimally invasive heart valve surgery) in our university hospital.

The key components of the ERAS model, as defined by the German Society for Thoracic, Cardiac, and Vascular Surgery (DGTHG) [8], are as follows:

1. The provision of interprofessional preoperative counselling.
2. It is recommended that patients engage in preoperative conditioning activities, including physical activity and improved nutrition through high-caloric supplementation.
3. The intraoperative and early postoperative management protocols should include early extubation and mobilization.
4. The early de-escalation of care is achieved through the activation of nursing care, intensive physiotherapy and the implementation of an individualized pain management plan.
5. The objective is to facilitate early hospital discharge directly to rehabilitation facilities.

To determine the effective implementation of ERAS and patient involvement, an ERAS coordinator, frequently a nursing professional (ERAS nurse), is designated to oversee treatment and act as a liaison for all relevant parties, particularly vital for ensuring early hospital discharge [6].

Since January 2021, ERAS has been implemented in our university hospital for minimally invasive heart valve surgery, offering two distinct ERAS approaches: an ERAS Light model, which primarily focuses on intraoperative and early postoperative care; and an innovative ERAS model, which applies ERAS perioperatively. Furthermore, a substantial number of patients received the standard of care that is currently the predominant approach in Germany. The three care models for minimally invasive heart valve surgery at our university hospital are described in detail in the methods section.

### Standard of care

The standard of care in minimally invasive cardiac surgery is as follows: patients for whom heart surgery is indicated are typically referred to cardiac surgery by private cardiologists or referring clinics. There, the patients receive an appointment for their heart surgery. Patients are usually admitted to the hospital one day before the surgery date and are routinely informed by the cardiac surgery and anesthesia departments on that day. The surgical procedure is typically performed within less than 24 h after the patient´s admission to the hospital. Following the surgical procedure, the patients are intubated and transferred to the intensive care unit (ICU), where they remain for one night before being transferred to the regular cardiac surgery ward in the absence of complications. A maximum of one physiotherapy session is provided per day in this setting. Postoperatively, with the patient’s consent, an application for rehabilitation measures is submitted by the social services or rehabilitation management. The waiting period for admission to an inpatient rehabilitation clinic is typically between two and three weeks. Consequently, patients are initially discharged to their place of residence before they commence rehabilitation at a designated facility.

### ERAS model light

The patients undergoing treatment in accordance with the ERAS model light receive a course of care that incorporates both conventional and innovative elements of the ERAS model. This model places an emphasis on intraoperative modified anesthesia and specific surgical details, with the objective of enabling patients to be extubated already in the operating room. In lieu of transfer to the intensive care unit (ICU), patients are relocated to the Intermediate Care Unit (IMC), given that while they do not necessitate intensive medical care, they do require intensive nursing care and continuous monitoring of their vital functions. The IMC represents a level of care situated between that of the ICU and the regular ward. It is optimal for patients to receive early mobilization from physiotherapists or nursing staff on the evening of the surgery. One first postoperative day, ERAS patients are transferred to the regular cardiac surgery ward. In this setting, early de-escalation measures are initiated, including the timely removal of catheters and drains. Furthermore, the objective is for patients to be discharged either to their place of residence or to a rehabilitation facility between the fifth and sixth postoperative days.

The ERAS light model is analogous to the innovative, perioperative ERAS model. However, the elements that are perceived by patients, such as interprofessional preoperative education and counseling, care by an ERAS nurse, and intensive physical therapy, are not included in this ERAS model.

### Innovative ERAS model

The principal elements of the innovative ERAS model were implemented in accordance with the recommendations set forth in the DGTHG publication and the INCREASE protocol. The perioperative ERAS model at our university hospital comprises the following components [8, 11].

A preoperative educational session, attended by professionals from various disciplines, is held in the clinic two to three weeks prior to the operation. These sessions comprise comprehensive information provided to patients by the cardiac surgery and anesthesia teams. Additionally, the physical therapy department provides information about the expected physical limitations after the surgery, the best ways to manage them, and the importance of postoperative movement for quick recovery. Moreover, the physical therapy team instructs patients in the exercises they should perform in preparation for the surgery. In a meeting with the ERAS nurse, patients are informed about the upcoming hospital routine, receive nutritional recommendations, and are provided with a high-calorie protein drink for the last ten days prior surgery. Subsequently, a psychosomatic consultation is conducted to reinforce positive expectations regarding the surgery (expectation-focused intervention) and develop coping strategies for potential adverse events or symptoms. Patients are instructed in relaxation techniques and are given the opportunity to express any concerns or fears they may have. This active patient involvement and education also includes providing a patient diary with educational content and exercise sheets for patients to complete. Additionally, individual goals and expectations are discussed with all specialties and recorded in the diary to keep track of them during the postoperative phase. Furthermore, these goals and expectations are regularly reviewed with the ERAS team.

The involvement of relatives both preoperatively and postoperatively is of significant importance. It is recommended that patients be accompanied by their relatives during the preoperative educational session. In the period between the educational session and hospital admission, both patients and their relatives are afforded the opportunity to contact the ERAS nurse or the psychosomatic specialist.

Patients are typically admitted to the hospital one day prior to the scheduled surgical procedure. On this day, in addition to a brief discussion with the cardiac surgery team, patients also have the opportunity to meet with the ERAS nurse and the psychosomatic specialist once more.

In accordance with the ERAS model, patients are extubated in the operating room and subsequently transferred directly to the intensive care unit (ICU), where they undergo an intensive physiotherapy and mobilization program. Immediately following the surgical procedure, the ERAS nurse serves as the primary point of contact and provides ongoing support for the patient. The initial early mobilisation by physiotherapy is conducted three hours postoperatively, with a subsequent session occurring three hours later. On the following morning, patients are transferred to the regular cardiac surgery ward, where they receive intensive care from the ERAS nurse until they are discharged. On the initial postoperative day, patients are provided with four sessions of physiotherapy, with two sessions per day subsequently administered on subsequent days. Furthermore, patients have access to psychosomatic support at all times. On a daily basis, interprofessional ward rounds are conducted, involving all treating specialists. Patients are actively included in their treatment. Furthermore, the ERAS nurse performs nursing rounds and provides assistance to patients in utilising the patient diary.

Patients are transferred directly from the hospital to a rehabilitation facility. The perioperative ERAS model considers participation in a rehabilitation programme to be an integral aspect of the process.

### Research gap

Although numerous studies have demonstrated the efficacy of ERAS protocols in a range of surgical procedures, there is a clear need for further research to optimise protocols for specific patient groups, including those undergoing cardiac or heart valve surgery [12]. It is essential to gain an understanding of patient expectations and acceptance if new healthcare models are to be successfully implemented [13]. Qualitative studies exploring patient experiences with ERAS in cardiac surgery are scarce, yet indispensable for understanding patient needs and improving the quality of care [14]. Furthermore, the perspectives of patients have been largely absent from the development of ERAS guidelines, which highlights a research gap that this study aims to address. The objective of this study is to qualitatively assess patient satisfaction across different care models for minimally invasive heart valve surgeries, with a view to providing insights for optimized care and future ERAS implementations.

This study comprises a qualitative survey and an evaluation of patient satisfaction across three care groups for minimally invasive heart valve surgeries at our university hospital (standard of care, ERAS model light, and innovative ERAS model). The study derives recommendations for optimized care and the implementation of future ERAS programs from the patients’ perspective. This gives rise to the following research questions: What are the essential elements of optimal care for patients undergoing cardiac surgery, and what are the key differentiating factors between various care models?

## Methods

### Study design & data collection

To address the primary research questions, a qualitative approach was selected. Qualitative research provides insights into complex phenomena and explores the relationships and patterns within participants’ experiences and perspectives. The objective is to generate new insights and hypotheses. In a novel field of research, it is of paramount importance to initially obtain profound insights into intricate matters, as opposed to primarily investigating causal relationships, which is the objective of quantitative research.

Semi-structured interviews were conducted with patients who had undergone minimally invasive heart valve surgery, across the three different care concepts. The interview was structured around the following categories of inquiry:

1. Preoperative preparation
2. Care and communication during the hospital stay
3. Participation and involvement of patients and relatives
4. Rehabilitation and post-hospital course

The translated interview guide is attached as supplementary material.

The study included patients who met the following criteria: 1. A minimally invasive heart valve surgery at our hospital approximately two to three months prior, 2. The capacity to comprehend the German language, 3. A duly signed informed consent form.

A purposive sampling method was employed to select study participants, aiming for the greatest possible variability. This ensured diversity in terms of disease course, age, and gender. No compensation was provided to participants for their involvement in the study.

Each patient was interviewed on a single occasion, approximately two to three months after their surgical procedure. This timeframe was selected to ensure a sufficient interval between the patients’ hospitalization and the interviews, allowing them to reflect on their experience while their memories were still fresh. The interviews were conducted by two trained researchers via telephone and were recorded using a dictation device.

The aim was to conduct interviews with approximately 30 patients, a number consistent with similar studies [12, 13, 15]. To ensure adequate representation of the three distinct care groups, approximately ten patients were included from each group. From a methodological standpoint, it is advisable to include as many patients as possible until saturation is reached. Considering this, the recruitment process continued until no new insights were gained from the interviews.

The potential participants were identified through the cardiac outpatient clinic. Patients were recruited at their scheduled follow-up visits, which took place two to three months post-surgery. Prior to enrolment, all participants provided written informed consent.

The interviews were conducted from 01. August 2022 to 01. August 2023.

### Analysis

The interviews were conducted via telephone, recorded and subsequently transcribed. The data was then subjected to a structured qualitative content analysis in accordance with the methodology proposed by Kuckartz (2020). The data analysis was conducted using the Qualitative Data Analysis (QDA) software MAXQDA, which was developed by Kuckartz (2020) and is particularly suited to the analysis of results using his approach.

The objective of Kuckartz’s structured qualitative content analysis is not to test existing hypotheses but to expand an existing theory with new concepts. Kuckartz’s approach is both deductive and inductive, thereby enabling the research question and predefined main categories to be adjusted in a dynamic manner [16].

In this research, the content of the ERAS model and the various stages of patient care were used as the basis for the deductive formation of the main categories. By meticulous examination and incorporation of these elements, specific categories were devised that reflect the pivotal elements of patient care and the tenets of the ERAS model. Concurrently, inductive coding permitted the emergence of novel themes from the data, thereby facilitating flexibility and openness to participants’ distinctive perspectives.

The initial author, who was not involved in the data collection process, undertook the analysis of transcripts, debriefings, and reflexive and observational notes.

### Ethics

This study was approved by the ethical committee of the Medical Faculty of the Ludwig-Maximilians-Universtität München on 07/13/2022 (Project Number: 22–0464). Participants gave written informed consent.

## Results

### Description of population

The data collection process was conducted over a period of 12 months, from August 2022 to July 2023. The interviews lasted between 20 and 60 min, depending on the depth of the responses provided by the participants. The recruitment process was concluded due to the attainment of data saturation, indicating that no new insights could be derived from the interviews. A total of 36 patients were interviewed, with 12 patients from each of three distinct care groups. The patients ranged in age from 28 to 75 years (mean age: 57 years, standard deviation 12.84). Of the total number of participants, 29 were male and seven were female. The most frequently performed procedure was mitral valve reconstruction, which was undertaken in 18 patients, while eight patients underwent aortic valve replacement. A comprehensive list of additional valve and aortic procedures can be found in Table 1.

**Table 1:** Description of study population

**No.**	**Group**	**Age**	**Sex**	**Surgery**	**IMC/ICU**	**Considerable events during postoperative stay**
1	ERAS +	56	m	DAVID	IMC	n/a
2	ERAS +	75	m	MV^a^ repair	IMC	n/a
3	ERAS +	35	m	AV^b^ repair	IMC	Second surgery
4	ERAS +	60	m	MV repair	IMC	Atrial flutter
5	ERAS +	39	m	AV repair	IMC	Pacemaker implantation
6	ERAS +	46	w	MV repair	IMC	n/a
7	ERAS +	60	m	MV repair	IMC	n/a
8	ERAS +	38	w	AV repair	IMC	n/a
9	ERAS +	57	m	AV replacement	IMC	Pacemaker implantation, retransfer from rehabilitation to hospital due to pericardial effusion
10	ERAS +	62	w	AV replacement	IMC	Atrial fibrillation
11	ERAS +	52	m	AV + AA^c^ replacement	IMC	Atrial fibrillation
12	ERAS +	54	m	MV repair	IMC	Pneumonia, atrial flutter
13	SC	71	w	MV repair	ICU	n/a
14	SC	48	m	MV repair	ICU	n/a
15	SC	64	w	MV repair	ICU	n/a
16	SC	62	m	AV replacement	ICU	n/a
17	SC	67	m	AV replacement	ICU	n/a
18	SC	66	w	MV repair	ICU	Pleural effusion, atrial flutter, infection of breast implant > > capsulectomy and implant removal
19	SC	50	m	MV repair	ICU	n/a
20	SC	65	m	AV replacement	ICU	n/a
21	SC	53	m	DAVID	ICU	Retransfer to hospital due to pericardial effusion > VATS
22	SC	46	w	MV repair	ICU	n/a
23	SC	67	m	AV replacement	ICU	Atrial fibrillation, puncture-worthy pleural effusion
24	SC	66	m	AV replacement	ICU	n/a
25	ERAS	71	m	MV replacement	ICU	Atrial flutter, bradycardic atrial fibrillation
26	ERAS	45	m	AA^c^ replacement	IMC	Second surgery; wound infection
27	ERAS	75	m	MV repair	ICU	n/a
28	ERAS	53	m	MV repair	IMC	Pleura effusion
29	ERAS	28	m	AV repair + AA^c^ replacement	IMC	Pericardium, Dressler syndrome
30	ERAS	67	m	MV repair	ICU	n/a
31	ERAS	76	m	TV^d^ repair	IMC	n/a
32	ERAS	57	m	MV repair	IMC	n/a
33	ERAS	70	m	MV repair	IMC	Impaired systolic left ventricular pump function
34	ERAS	68	m	AV replacement	IMC	Left bundle branch block immediately postoperative
35	ERAS	28	m	MV repair	IMC	n/a
36	ERAS	55	m	MV repair	IMC	n/a

^a^Mitral valve

^b^Aortic valve

^c^Ascending aorta

^d^Tricuspid valve

A total of 21 patients were transferred to the IMC directly following surgery, while 15 were admitted to the ICU. Complications were observed in 14 patients. It is notable that these are largely typical consequences of minimally invasive valve procedures, such as pleural effusions or cardiac arrhythmias, which are not clinically significant. Nevertheless, it is possible that these events may be associated with feelings of anxiety or uncertainty for patients. Therefore, even mild complications are included in the table.

In the table, abbreviations are employed to categorize patients into three distinct care groups (ERAS +  = innovative ERAS model, ERAS = ERAS model light, and SC = standard of care)

### Data

**Fig. 1:**
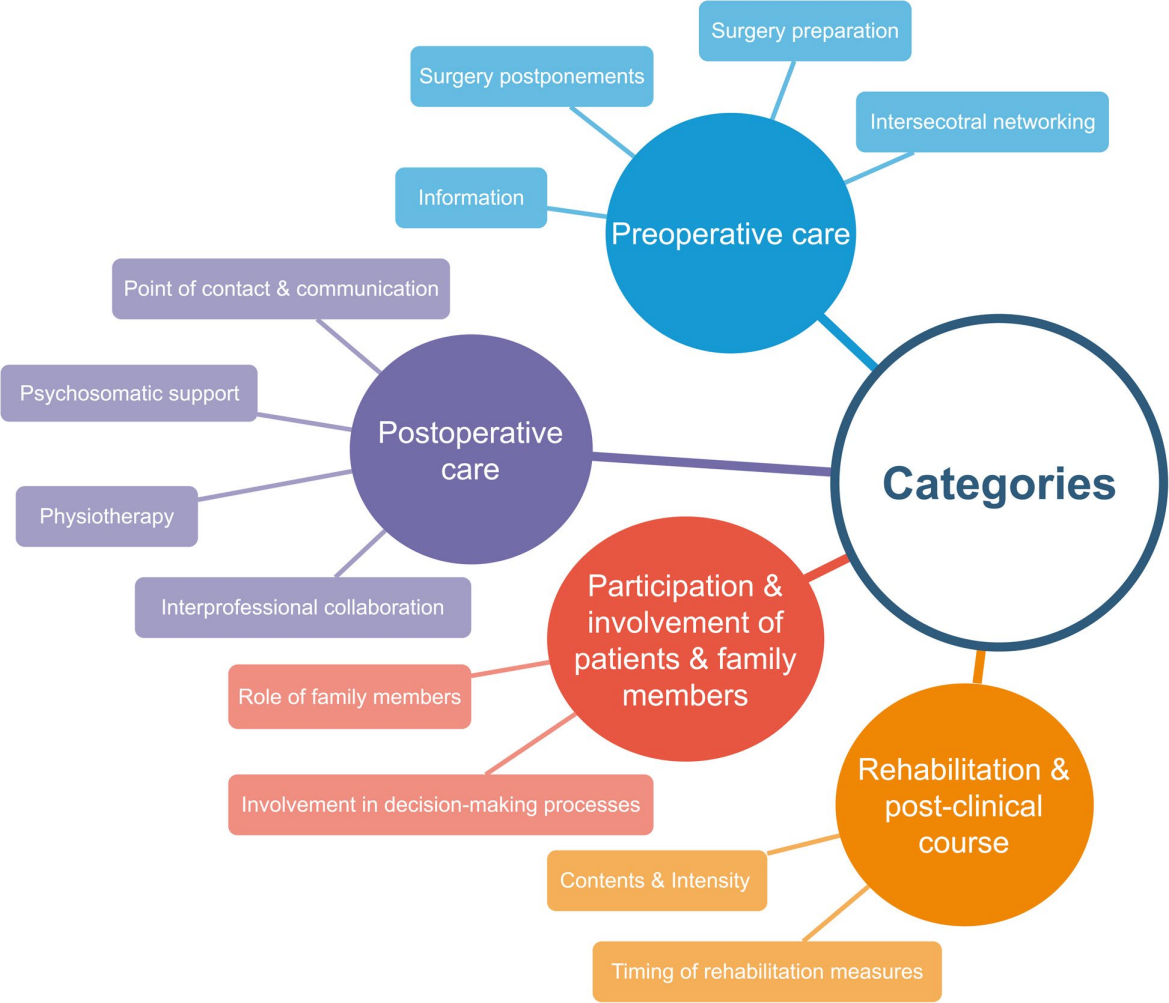
Category system

The findings of the interview analysis are presented in the following section. The analysis yielded four principal categories. The categories identified were as follows: (1) Preoperative Care, (2) Postoperative Care, (3) Participation and Involvement of Patients and Family Members, and (4) Rehabilitation and Post-Clinical Course. Subsequently, subcategories were formed within the aforementioned main categories. These are illustrated in Fig. 1 and will subsequently be described and exemplified with quotations. Each quotation is accompanied by an abbreviation indicating its affiliation with one of the three care groups: ERAS + , ERAS light, or SC.

#### Preoperative care

The first main category describes the preoperative care of the patients. This category is comprised of four subcategories: a) information, b) surgery postponements, c) surgery preparation, and d) intersectoral networking.

##### a) Information

The ERAS + cohort demonstrated consistent satisfaction with the quality and content of their preoperative information sessions, which occurred approximately two to four weeks prior to surgery.

“Yeah, I also found it good in the sense that different professional groups were present, you got to know the people a bit, and you received quite a bit of explanation in advance about what will happen, how it will go.” (ERAS+)

The interprofessional preoperative discussions, which encompassed surgical and anaesthesiological information, as well as consultations with ERAS nurses, physiotherapy, and rehabilitation management, in addition to with psychosomatic and mental preparation, were unanimously regarded as beneficial and enriching. A number of patients indicated that these discussions assisted them in more effectively managing their anxiety about the surgery, thereby enabling them to approach it with greater confidence. The timing of these discussions was also deemed appropriate, allowing sufficient time for patients to mentally prepare for the surgery.

“That was good, good preparation. It really helped. I was really scared of the surgery. But this preparation really helped me. Psychologically as well. And I could contact the lady [psychosomatic therapist] again before the surgery. That was good because sometimes I was really scared. It calmed me down again.” (ERAS+)

The majority of ERAS light patients expressed satisfaction with the surgical information they received, which typically included details pertaining to the surgical procedure and anaesthesia. This information was typically provided one day prior to the surgery. A number of patients indicated that they had received information about the surgery from previous examinations or discussions, both within the clinic and with their outpatient cardiologist. The ERAS light patients often demonstrated a paucity of inquiries during the information sessions, which suggests a lack of knowledge and familiarity with the subject matter. Some ERAS light patients proposed that the provision of supplementary information could prove beneficial.

“So I felt sufficiently informed. I have to admit, I didn’t want to know so much about what exactly they do. Because I was naturally afraid of the surgery. [...] I didn’t have many questions about it, I have to say. I just let it come to me.” (ERAS light)

“No, I didn’t ask much there. Because I have no idea about medical things. What should I ask?” (ERAS light)

Some SC patients reported feeling inadequately informed, citing the lack of pre-surgical information as a significant factor. These patients had only received the information session the day before surgery, with minimal or no information provided beforehand. Although the process was comparable to that undergone by ERAS light patients, there was a discrepancy in the perception of it between the two groups. As with ERAS light patients, a significant proportion of SC patients placed considerable trust in the medical professionals and demonstrated a reluctance to engage in decision-making or pursue further information.

“For me, it was actually sufficient. So I couldn’t say anything about it because I’m not a medical professional. You would sometimes inquire more [if the information session were earlier], but you’re also glad when it’s finally over and you understood it. And that’s actually the most important thing.” (SC)

##### b) Surgery preparation

Patients who were enrolled in the ERAS + program were actively engaged in preparing for their surgery following preliminary discussions. They reported modifying their diet and fluid intake, maintaining regular physical activity, abstaining from alcohol, and, in some cases, quitting smoking. Additionally, they had the opportunity to mentally prepare for the surgery and to utilize relaxation and distraction techniques that they had previously learned during their preliminary discussions. Moreover, they reported feeling adequately prepared upon admission to the hospital the day before the scheduled procedure.

“Um, the surgery preparation was particularly helpful for me: Actually, what can I do to optimize the surgery process - with nutrition, with exercise, with exercises.” (ERAS+)

“And I was just really well-prepared and informed about everything that was coming my way.” (ERAS+)

Both ERAS light and SC patients demonstrated a lack of preparation for the surgery, with few having a clear understanding of the steps they could take to adequately prepare. A minority of SC and ERAS light patients reduced their alcohol consumption in the period preceding the surgical procedure. The interviews revealed a passive attitude towards surgical preparation. In the majority of cases, both ERAS light and SC patients were unaware that preparing for surgery could positively impact their postoperative recovery. They placed complete trust in the medical professionals in charge.

“I wouldn’t even know how I could have prepared myself for anything.” (SC)

##### c) Surgery postponements

It was frequently reported by both ERAS light and SC patients that their surgery dates were postponed at short notice (on multiple occasions in some cases), which is a situation that can occur across all care groups. Some patients reported instances of poor communication and inadequate information, which they perceived as a source of frustration and a lack of transparency. Patients who were aware of the rationale behind the postponement of their surgery dates (e.g., due to emergencies or strikes) demonstrated a greater capacity to accept these postponements, exhibiting an understanding of the circumstances and the medical staff involved. It is notable that no instances of surgery postponements were reported among the ERAS + cohort.

“The communication, that didn’t work, it didn’t go well. Because I was postponed so many times and that was poorly communicated.” (ERAS light)

##### d) Intersectoral networking

A significant number of ERAS light and SC patients expressed considerable trust in the recommendations of their outpatient cardiologists. Patients who received care through the ERAS + program rarely referenced their referring doctors or clinics.

“What should I do with [more information before the surgery]. My cardiologist determined that something is really wrong with me, and I have to follow the medical advice. I’m not a physician.” (ERAS light)

#### Postoperative care

The second main category pertains the postoperative care of patients. This category comprises four subcategories: a) point of contact and communication, b) psychosomatic support, c) physiotherapy, and d) interprofessional collaboration.

##### a) Point of contact and communication

ERAS + patients reported high levels of satisfaction with the information they received and the dedicated point of contact provided by the ERAS nurse, whom they trusted and who furnished them with all necessary information. The ERAS nurse played a pivotal role in fostering a sense of support and guidance for patients.

“I think it was just a huge support for me. You didn’t feel so alone. And if a question came up, you could clarify it immediately with her [the ERAS nurse].” (ERAS+)

“Throughout the whole process, I didn’t feel like just a number but as a patient who was being treated and cared for. And that’s really something very important and good for me.” (ERAS+)

ERAS light patients did not have an ERAS nurse involved in their care, however, they expressed overall satisfaction with the quality of care and the information flow. The respondents identified nursing staff and ward doctors as their primary points of contact, which presented challenges due to the rotating medical staff.

“You had to follow up to get more information.” (ERAS light)

SC patients frequently encountered delays and lapses in communication, often feeling insufficiently informed, often reporting a lack of sufficient information, which in turn gave rise to feelings of uncertainty. A desire was expressed for the provision of a listening presence and a source of support during periods of illness.

“I didn’t know what I was allowed to do. Can I shower if I feel unwell? I just didn’t know, and it was tough in that moment.” (SC)

“I would have liked someone who could say, ‘Ah, you have this and that, it’s quite normal. Don’t worry.’ Or someone who could tell you if it’s normal to not feel well.” (SC)

In all groups, the majority of patients expressed a desire for feedback on the surgical outcome. Furthermore, many patients indicated a wish to meet their surgeon, either before or after the surgery. These encounters were described as reassuring and as contributing to the patients´ confidence in the procedure.

“It would have been nice to have a decent conversation after the surgery about what was actually done. Unfortunately, that didn’t happen. An explanation a day or two afterwards. Something like that was done in the intensive care unit. But unfortunately, I can’t remember that.” (ERAS light)

##### b) Psychosomatic support

The ERAS + patients received psychosomatic support both pre- and postoperatively, which they uniformly perceived as beneficial. All patients in the ERAS + group participated in a psychosomatic discussion on the day preceding the surgery. Subsequent to the surgery, a minimum of one discussion was conducted, with the possibility of additional meetings if required. In certain instances, this occurred on a daily basis. Patients experiencing elevated anxiety levels were more likely to utilize the psychosomatic support offered in the postoperative period.

“Exactly, we actually did an exercise in autosuggestion. It wasn’t completely new to me, but it was good. [...] What I also liked was that we made a list. What could happen, like pain, anxiety, whatever. And then we always tried to find possible solutions or behaviors to counteract them. That usually helped me.” (ERAS+)

“The psychosomatic aspect was very helpful. Like how the course could be. That there could be lows after the surgery, but that it’s also quite normal. That it usually gets better continuously. But also to see the whole thing positively.” (ERAS+)

Neither ERAS light nor SC patients received psychosomatic support. However, the majority of respondents indicated that such support was beneficial for specific patient groups. The necessity of such support was perceived as being contingent upon the individual rather than being universally applicable. Some ERAS light and SC patients reported feelings of preoperative anxiety and apprehension about the forthcoming surgery. In the postoperative period, the occurrence of episodes of low mood was recounted on multiple occasions. Additionally, some patients reported experiencing mental limitations several weeks after their surgery.

“I didn’t think that after the surgery things would be so tough psychologically.” (SC)

##### c) Physiotherapy

The ERAS + cohort was mobilized for the first time three hours postoperatively. A maximum of four physiotherapy sessions were conducted on the first postoperative day, with two sessions scheduled daily thereafter. Some patients who underwent the ERAS + program reported that the intensive physiotherapy was both tiring and motivating. The guidance provided by physiotherapists enabled ERAS + patients to gain a deeper understanding of the permitted range of movements and exercises, which in turn facilitated a more expedient recovery.

“It’s an advantage when [...] from the second or third hour after the surgery, you’re already accompanied by physiotherapists. Where you might be unsure – ‘What can I do now?’ or ‘Can I walk, get up, whatever?’ They always encouraged me to do it. Yes, and then I dared to do it.” (ERAS+)

Some ERAS light and SC patients underwent minimal physiotherapy, with some reporting no sessions at all. The patients did not perceive this negatively; either they believed it was too early for such activities or they preferred to rest.

“I mean, just after the surgery, I don’t know what I could have done anyway.” (ERAS light)

##### d) Interprofessional collaboration

The concept of interprofessional collaboration was interpreted in a variety of ways by patients. The majority of ERAS + patients expressed high levels of satisfaction with the collaborative approach adopted by the ERAS team. The only instance in which the collaboration between nursing staff and medical personnel on regular wards was deemed unsatisfactory was in the occasional criticism levied against it.

“I felt that they worked very hand in hand. As I said, nursing staff and then physiotherapists with the ERAS nurse and psychosomatics.” (ERAS+)

“Sometimes, there probably could have been a bit more communication between doctors and ward staff.” (ERAS+)

A lack of effective communication between different professional groups was identified as a concern by both SC and ERAS light patients.

“And I think the coordination wasn’t good there. I don’t know who should coordinate with whom. Anyway, if I hadn’t said anything, I wouldn’t have gotten the pills.” (SC)

#### Participation and involvement of patients and family members

The third main category comprises the participation and involvement of patients and family members, which can be divided into two subcategories: a) involvement in decision-making processes and b) the role of family members in the care process.

##### a) Involvement in decision-making processes

ERAS + patients reported feeling extensively involved in all processes of their care through comprehensive preoperative discussions, which they perceived as positive. This enabled them to gain a deeper comprehension of their illness trajectory and to become fully informed, thereby empowering them to assume responsibility for their own health. During their hospitalisation, they felt actively integrated into their treatment, primarily through the ERAS nurse and interprofessional care. They did not feel that decisions were made without their involvement. In general, ERAS + patients experienced few postoperative events that were surprising or unexpected. In the event of complications, patients were able to comprehend the reasons and were supported by the ERAS nurse and psychosomatic support.

“Yes, so both the woman from psychosomatics and the ERAS nurse were there almost daily [...] and took care of me, talked to me, discussed various things, and encouraged me. So that was very good. Especially when you maybe had a low point or something, where they helped you over it a bit and encouraged you.” (ERAS+)

The ERAS light and SC patients demonstrated a more passive approach to their treatment trajectory. Notably, during the preoperative clarification phase, patients demonstrated minimal active involvement (see Results – Preoperative Care). Throughout their treatment, ERAS light and SC patients continued to rely heavily on their doctors’ decisions, with minimal opportunity or perceived benefit in assuming an active role in their treatment trajectory.

“I: Did you feel involved in your treatment?

B: Not really. What, what can you do there? Except fiddling around with the lung exercise machine. We know ourselves - taking medication – that’s about it. So, there’s not much more to it.” (SC)

During regular ward rounds, patients undergoing the ERAS light and SC pathways reported a lack of active involvement. As illustrated in point 2, SC and ERAS light patients reported instances of inadequate communication. The patients reported feelings of uncertainty.

“So after the operation, the big round comes - they don’t talk to you, they talk about you.” (SC)

##### b) Role of family members in the care process

The role of family members in the care of patients is a significant one, regardless of the patient group in question. In all forms of care, patients are permitted to bring their family members for preoperative explanations. For patients enrolled in the ERAS + program, this was explicitly highlighted and patients were advised to bring a family member or friend with them. All patients with family members present reported that this was beneficial. Primarily, family members were able to pose questions, and secondly, they could serve as a memory aid following the conversation. For patients enrolled in the ERAS + program, comprehensive explanations were also beneficial for their family members, as this enabled them to better cope with the circumstances and provide effective support to the patient.

“What I have to say was really great was that my husband could be there too, because in such moments one is often a bit excited or forgets some things to ask, and when the partner is there, they can actually step in for you or they have other questions.” (ERAS+)

In the postoperative period, there was minimal variation in the involvement of family members across different forms of care. Across all forms of care, patients identified family support as a crucial and beneficial aspect. Similarly, a proportion of patients across all forms of care indicated a preference for reduced family involvement. Furthermore, family members of ERAS + patients proactively contacted the ERAS nurse and the psychosomatic contact person. Some patients indicated that their surgery had a significant psychological impact on their family members.

“And also that I didn’t have anyone to talk to in that situation, not even my husband. So, that was missing for me.” (SC)

#### Rehabilitation and post-clinical course

The final main category encompasses rehabilitation and the post-clinical course, consisting of two subcategories: a) timing of rehabilitation measures and b) contents & intensity of rehabilitation.

##### a) Timing of rehabilitation measures

Patients undergoing the ERAS + pathway were typically transferred directly from the hospital to rehabilitation facilities. In the other two forms of care, the waiting period ranged from a few days to several weeks. A minority of patients surveyed did not engage in any rehabilitation measures.

The majority of patients from all three forms of care perceived the sequence of events they experienced to be beneficial. ERAS + patients believed that being swiftly transferred to rehabilitation was beneficial, as it avoided a break in their care. One advantage of direct transfer was uninterrupted medical and professional care, which provided ERAS + patients with security after early hospital discharge. Family members also perceived this as a positive outcome.

“No, for me, it was good to continue right there. I think if I had been at home for a few days or maybe longer, I wouldn’t have known what to do. That would have been a break.” (ERAS+)

“So, for me and primarily for my husband and other relatives, it was really great. Optimal, because in the first few days of rehab, situations often arose [...] where you think: ‘Wow, is this normal?’ So, if I had been at home, I would have been worse at handling it. And indeed, my husband too, because in that case, he also doesn’t know what to do.” (ERAS+)

In particular, the SC patients who were required to wait several weeks before commencing rehabilitation often perceived this period as an opportunity to recuperate from hospitalization and to rest before resuming their rehabilitation program.

“No, the timing was good for me. Because I have to say, when I got home, I kind of fell into a hole - probably didn’t move enough or something. And that’s why I was actually okay with the start of rehab taking that long.” (SC)

A number of ERAS light and SC patients elected to decline participation in rehabilitative programs. A variety of reasons were provided. For instance, one patient indicated that they lacked the requisite strength to engage in rehabilitation activities, whereas another expressed a preference for conducting their rehabilitation at home. At the time of the survey, some SC and ERAS light patients had not yet received a rehabilitation appointment, typically eight to twelve weeks postoperatively.

“Because I was too weak for rehab. I said, ‘No, I can’t go.’ If I can’t walk ten steps, that’s no use.” (SC)

##### b) Contents & intensity of rehabilitation

In general, the majority of patients reported that their rehabilitation stay was meaningful and that it had a positive impact on their recovery. All patients who had undergone the ERAS + program were satisfied with the stationary rehabilitation stay that they had undergone.

“Overall positive. I have to say, I was practically on rehab after four days. At that moment, I actually thought, ‘Oh my God, this is all too much.’ And I couldn’t do anything - but then after two to three days of rest and getting used to it, [...] with smaller training sessions, I actually saw compared to other patients that I was already quite far in the recovery process.” (ERAS+)

Furthermore, SC and ERAS light patients were largely satisfied with the rehabilitation they received. The discrepancy in preoperative information provided to ERAS light and SC patients, in comparison to that provided to ERAS + patients, was partially rectified in the rehabilitation clinics that were visited post-hospitalization.

“But actually, afterwards in rehab, I had a good doctor who explained everything from start to finish again, what was done. And then I knew very well what was going on with me.” (ERAS light)

The majority of patients were able to resume independent management of their daily lives following the completion of the rehabilitation program. For patients who received the ERAS + pathway, this was sometimes the case at an earlier stage, as they were able to commence rehabilitation immediately following their hospital discharge.

“I wouldn’t have thought that it would happen so quickly, that I would be back on my feet and have most of my daily routine back.” (ERAS light)

## Discussion

The objective of the present study was to evaluate patient perspectives on various care processes in minimally invasive cardiac surgery and to derive insights into the optimal care process from the patient’s viewpoint. It was found that comprehensive preoperative education and seamless communication throughout the entire perioperative care process are fundamental elements for patient satisfaction and an optimal care process. Patients who received care according to the ERAS + model expressed greater satisfaction with their care than patients who received care according to the SC or ERAS light models.

### Preoperative patient education

The findings highlight the importance of preoperative education as a pivotal phase in the care process, enabling patients to actively engage in their recovery. Comprehensive preoperative education empowers patients by providing them with detailed information in a timely manner, enabling them to take proactive steps to optimize their health before the surgery. In particular, patients who underwent the ERAS + approach benefited significantly from this initiative, which commenced several weeks prior to surgery. This contrasts with the experiences of patients who underwent SC and ERAS light, who appeared to be less informed and prepared. This proactive engagement among ERAS + patients continued into their postoperative care, in contrast to the more passive attitude observed among ERAS light and SC patients, who relied more on medical guidance. These findings illustrate the beneficial effect of patient empowerment, facilitated by the ERAS + model, on patient outcomes and satisfaction.

The extant literature substantiates the beneficial impact of preoperative patient empowerment on a range of postoperative outcomes, including pain reduction, decreased opioid consumption, anxiety alleviation, enhanced wound healing, improved self-care ability, and increased self-efficacy [17–21]. Patient education is of paramount importance in fostering patient empowerment [22].

In light of these findings and the current literature, it is strongly recommended that comprehensive preoperative patient education be made available to all cardiac surgery patients [23]. It is recommended that these sessions occur at least two weeks prior to surgery, thus allowing patients to proactively prepare for their surgery [24].

### Patient education and empowerment

The literature identifies nursing professionals as pivotal figures in the processes of patient education and empowerment [25]. In the ERAS protocol, ERAS nurses, in collaboration with psychosomatic specialists, play an important role in fostering patient empowerment. A patient-centered approach is fundamental to the successful implementation of patient empowerment [26], which is a core tenet of the ERAS protocol (German Society for Thoracic, Cardiac, and Vascular Surgery, 2021). Therefore, the perioperative ERAS protocol represents an optimal framework for achieving patient empowerment [11].

However, comprehensive patient education and empowerment do entail resource costs in terms of personnel and time. Nonetheless, Burgess et al. [27] posit that the initial investment of time may yield to long-term benefits, namely enhanced patient co-management in care processes, thereby contributing to a robust and cost-effective healthcare system. It has been demonstrated that the implementation of a structured preoperative patient education program can result in a reduction in the length of hospital stay, along with a decrease in complications both during and after discharge [28]. Moreover, active patient involvement in their care can result in more effective and appropriate resource allocation [29, 30]. In particular, shorter hospital stays and treatment in IMCs rather than ICUs have been associated with cost reductions within the context of ERAS protocols [31].

Familiarizing patients with the roles of various healthcare professions during preoperative education not only enhances their understanding of the care process but also reinforces the competencies and importance of all involved professionals [32, 33]. Furthermore, an enhanced appreciation for all healthcare professions has the potential to reinforce the positive workplace climate and staff satisfaction [34, 35].

### Involvement of family members

It is of the utmost importance that family members are actively involved in the care process as a support system for patients, as this has been proven to be crucial in numerous studies. The results indicate that, in particular in the postoperative period, greater involvement and support for family members could be beneficial. Providing patient- and family-centered care is essential, as it is imperative to meticulously consider the individual needs of each patient. The extant research on family-centered care is primarily focused on the ICU setting [36–38].

A variety of strategies may be employed to facilitate the involvement of family members in non-ICU settings. These include the designation of dedicated points of contact within the clinic, such as ERAS nurses or psychosomatic counsellors, the provision of psychological counselling, the organization of support groups for family members, and the dissemination of informative materials tailored to their needs. It is recommended that the content include advice on post-hospital discharge patient care and the role of family members in supporting the patient’s recovery at home.

### Psychosomatic support

The psychosomatic support was met with a uniformly positive response. However, it should be noted that the individual needs for psychosomatic support varied across all groups. SC and ERAS light patients frequently reported emotional changes following surgery that they found challenging to comprehend, indicating that psychosomatic support may have been advantageous.

Heart surgery can induce anxiety and psychological stress in both patients and their families [23, 39, 40]. It has been demonstrated that psychological interventions and preoperative education can have a beneficial effect on anxiety and depression levels in patients undergoing heart surgery [23, 40, 41]. It is therefore imperative that psychosomatic counselling and mental preparation are incorporated into the preoperative patient education program. Furthermore, it is recommended that psychosomatic support be made available to all cardiac patients, both pre- and postoperatively. It is important to adapt the extent and content of the support provided to the specific needs of the individual in question [42].

### Physiotherapy

The disparate perceptions of physiotherapy as evidenced in our results highlight the necessity for a detailed examination of patient needs and expectations regarding rehabilitative measures. This approach guarantees that all patients receive the most efficacious support to facilitate recovery and achieve long-term results. All ERAS + patients reported that they found the provision of intensive physiotherapy support to be beneficial. Conversely, patients in the ERAS light or SC groups occasionally reported a lack of confidence in participating in hospital-based physiotherapy due to uncertainty about the permitted range of early postoperative movements. This discrepancy in patient perception underscores the potential benefits of providing education and support in a way that empowers patients. The postoperative self-assessment and perception of patients appear to be significantly influenced by the preoperative education they receive.

Intensive postoperative physiotherapy has been demonstrated to significantly enhance the healing process, promoting physical activity and restoring confidence in postoperative recovery. This enables a more rapid recovery, even in patients who might otherwise adopt protective behaviors. Furthermore, early physical activity has been demonstrated to reduce the length of hospitalization [43].

Based on these findings, it is recommended that all cardiac surgery patients receive intensive physiotherapy support. Group physiotherapy may offer a solution to the challenge of increasing the number of therapy sessions, despite the limitations of personnel resources. The positive feedback from ERAS + patients suggests that a patient-centered approach to care is of paramount importance in this context [44, 45].

### Communication

Effective and seamless communication was identified as a critical factor in patient satisfaction. The presence of ERAS nurses in the care of ERAS + patients facilitated effective communication, providing a trusted point of contact and enhancing their sense of security. In contrast, ERAS light and SC patients were not provided with a designated contact person or confidant, which occasionally resulted in communication challenges and frustration. The provision of clear information facilitates patient comprehension of hospital procedures and the subsequent recovery process. The presence of a designated ERAS nurse offers numerous advantages for patients, while simultaneously alleviating the workload of other healthcare professionals [7, 46, 47]. Moreover, an ERAS nurse serves as a liaison between all parties involved in the patient’s care, facilitating interprofessional communication [47, 48].

In light of the findings of this study, it is strongly recommended that an ERAS nurse be appointed in the cardiac surgery department. Furthermore, interprofessional rounds represent an efficacious instrument for enhancing communication and collaboration among healthcare professionals [49, 50]. Such professionals facilitate patient involvement in treatment decisions and provide opportunities for family members to be included [51]. All patients expressed a desire for postoperative feedback from the surgical team. One potential solution is the standardized implementation of brief surgical feedback discussions before hospital discharge. However, there is a paucity of literature on this approach.

### Limitations

The study is constrained by its modest sample size, which constrains the scope for generalizing the findings to a more extensive population. A larger and more diverse sample would facilitate a more comprehensive understanding of patient perspectives. However, the study continued until data saturation was achieved within the existing sample, and no further insights could be gained in the context of the current research design. Furthermore, the gender distribution within the sample is not balanced, partly due to a higher proportion of male patients within the surveyed patient population.

The data are based on self-reported patient experiences, which may be subject to bias, including recall distortions. The interviews were conducted approximately two to three months after surgery, which may not have been a sufficiently long period of time to capture all aspects of postoperative care. A longer data collection period could facilitate the identification of long-term effects associated with different care concepts. However, this approach may also increase the likelihood of introducing biases.

The study’s single-center design may limit the generalizability of its findings to other healthcare settings. A potential limitation of the study is the possibility of selection bias, which could affect the representativeness of the sample. It is possible that patients who agree to participate may differ in certain characteristics from other populations, which could impact the generalizability of the findings.

Telephone interviews, while practical and accessible for participants, may have limitations in capturing non-verbal cues and nuances of communication. This method was selected for its convenience and flexibility, which facilitated higher participation rates and a diverse sample.

### Future research

Future research could benefit from the involvement of a range of stakeholders in order to gain a more comprehensive understanding of the care process. Furthermore, the active involvement of patients in the development of care concepts and enhanced recovery after surgery (ERAS) programmes could ensure that their needs and preferences are adequately addressed.

Quantitative studies could complement the qualitative findings of this research. Specifically, large-scale quantitative studies could help validate the insights gained from qualitative research by providing statistical evidence on the effectiveness of different care models and ERAS programs. Such studies should focus on measuring specific outcomes such as patient satisfaction, recovery times, and the incidence of postoperative complications across various care models.

Furthermore, implementation research may facilitate the identification and resolution of challenges and barriers inherent to improvement measures. Furthermore, longitudinal studies could be employed to monitor the long-term effects of care changes on patient outcomes, satisfaction, and well-being.

## Conclusion

In conclusion, this study contributes to the existing body of knowledge regarding perioperative care for cardiac patients. Insights of considerable value were obtained by comparing three distinct care concepts from the perspective of the patients. The provision of preoperative education seems to establish the foundation for patients’ subsequent behavior and expectations regarding their treatment. Topics such as physical activity, nutrition, and mental health are highlighted as particularly relevant in this context.

The active involvement and engagement of patients and their families throughout the treatment process appears to support improved postoperative care, more intensive physiotherapy, mental support, and a faster recovery. It is essential that the entire care process is characterized by a seamless flow of information. The presence of a dedicated point of contact, such as an ERAS nurse, has been demonstrated to have a beneficial impact on patient well-being.

The study suggest that integrating interdisciplinary preoperative patient education and psychosomatic support within ERAS concepts could be beneficial for improving outcomes for a diverse range of cardiac surgical patients. Future quantitative research might further explore these concepts to better understand their effectiveness and applicability across diverse patient populations.

## Supplementary Information

Supplementary Material 1.

## Data Availability

The datasets generated and/or analysed during the current study are not publicly available due to data protection regulations and privacy concerns for the participants involved but are available from the corresponding author on reasonable request.

## Abbreviations

ERAS: Enhanced Recovery After Surgery
ICU: Intensive Care Unit
IMC: Intermediate Care Unit
SC: Standard of Care
