# Supplementary material for: Patient perspectives on health care models in cardiac surgery: a qualitative evaluation
Source: BMC Health Serv Res. 2024 Oct 30;24:1309. doi: 10.1186/s12913-024-11791-6 (PMC11524004; doi:10.1186/s12913-024-11791-6)
Supplement: Supplementary file 1 — Supplementary Material 1. [file 12913_2024_11791_MOESM1_ESM.docx]

**Interview Guide**

*English translation, originally conducted in German.*

**Introduction for the Interviewer:**

Begin with an introduction, providing an overview of the research project and its objectives:

The study focuses on three different care models for patients undergoing minimally invasive heart valve surgery

- **Standard of Care without ERAS (Enhanced Recovery After Surgery)**
- **Innovative, perioperative ERAS Care (ERAS+):**
  - Interprofessional preoperative consultation.
  - Preoperative conditioning of patients concerning physical activity and high-calorie nutrition.
  - Specific protocol for intraoperative management and early postoperative care.
  - Early de-escalation through proactive nursing care.
  - Intensive physiotherapy and personalized pain management.
  - Early discharge directly into rehabilitation.
  - **Key Characteristics:**
    - Cross-sector collaboration.
    - Interprofessional approach.
    - Care provided by an Advanced Practice Nurse (APN) as the ERAS Nurse.
    - Close psychosomatic support before, during, and after heart valve surgery.
- **Intraoperative and Early Postoperative ERAS (ERAS Light):**
  - Excludes interprofessional care before and after surgery.
- **Research Questions:**
  - What are the essential elements of optimal care for patients undergoing cardiac surgery?
  - What are the key differentiating factors between various care models?
  - What are the benefits, acceptance, and satisfaction levels among the affected groups?
  - What potential improvements can be identified from the perspective of all stakeholders?
- **Benefits of the Study:**
  - Enhancing future patient care and optimizing processes within this care pathway.
  - Establishing the optimal care process for patients with heart valve conditions as a standard and extending it to other clinics.
  - Strengthening interdisciplinary and cross-sectoral collaboration across hospitals, preventing gaps in care.
  - Elevating the role and relevance of the nursing profession.
- **Data Collection, Storage, and Usage:**
  - Semi-structured interviews.
  - Duration: Approximately 30 to 60 minutes.
  - Recorded using an audio device, with deletion after transcription.
  - Transcripts will be anonymized (no names, addresses), ensuring no personal identification. Transcripts will not be published if there is any possibility of identifying the individual.
  - Conversation logs will be maintained.
  - Collected personal data will be subject to confidentiality and data protection laws.
  - Right to information.
  - Data will be archived for 10 years based on GDPR guidelines.
- **Voluntary Participation and Right of Withdrawal:**
  - Participation and consent are voluntary.
  - Right to withdraw consent at any time.

**Telephone Interview Process:**

1. **Introduction:**
   - Confirm if the interviewee has time to talk.
   - Mention that this is a patient survey, and they may have received information during their outpatient visit.
2. **Explain the Purpose of the Survey:**
   - Emphasize that it is an opportunity for patients to share their opinions, subjective experiences, and provide feedback on their hospital care.
   - Highlight both positive aspects and areas for improvement.
3. **Ask if They Would Like to Participate:**
   - If they agree, inquire if they would prefer to schedule a separate appointment and send additional information.
   - Ask if they can participate immediately if their informed consent has already been obtained. (If not, informed consent has to be obtained, before you can continue with the interview.)
4. **Oral Briefing:**
   - Review information from the provided document.
   - Address any questions they may have.
   - Clarify that there are no right or wrong answers; the focus is on their personal experiences.

**Notes on the Questionnaire:**

- The sequence of questions can be adjusted. The first question of each category is the main question. The following sub questions can be used, if necessary, to get more detailed answers.
- Follow up on brief or unclear responses.
- Show empathy towards patients.

**Interview Questions (Vary by Group):**

1. **ERAS+:** You've had minimally invasive heart surgery, correct? Do you know what was different about your treatment compared to patients not in the perioperative ERAS program? **ERAS ligth:** You've had minimally invasive heart surgery, correct? Do you know if anything was done differently compared to the standard of care? **SC:** You've had minimally invasive heart surgery, correct?
2. **Surgical Consultation:**
   - Did you have a preoperative consultation about your surgery? Who conducted it (e.g., a doctor from the clinic or your cardiologist) and when did this take place, and how was the consultation for you?
   - *Did you feel sufficiently informed?*
   - *Were you able to ask all your questions?*
   - *What was particularly helpful?*
   - *How did you feel after the consultation?*
   - *Would you have liked more time to prepare for the surgery?*
   - *How did you find the timing of the consultation?*
3. **Preparation for Surgery:**
   - How did you prepare for the surgery? (Diet, exercise, gathering information, tasks, conversations)
4. **Mental/Emotional Well-being Before and After Surgery:**
   - How did you feel mentally before and after the surgery?
   - *Were you anxious about the surgery?*
   - *Did you feel secure and well cared for?*
   - *Did you feel that surgery was the right decision?*
5. **Hospital Stay Experience:**
   - How did you find your overall stay at the hospital, both before and after surgery up to rehabilitation?
   - ***ERAS+:*** *How helpful was the ERAS Nurse?*
   - ***ERAS+:*** *How helpful was the psychosomatic counseling and support?*
   - *What did you find helpful or supportive during your stay?*
   - *What aspects did not go well?*
   - *Did you miss anything during your stay?*
   - *Was there anything you found unnecessary or unhelpful?*
   - *Which processes were particularly time-consuming? Could any be more efficient?*
6. **Overall Care Process:**
   - Was there anything you wish had been included in your care?
7. **Involvement in Care Process**
   - Did you feel involved in your treatment process? Did you feel decisions were made together with you about the next steps?
   - *Can you describe this in more detail?*
   - *Which professionals contributed to this?*
   - *Were you able to contribute to your care process?*
8. **Information Flow:**
   - How did you perceive the flow of information? Did you always know what would happen next?
   - *Did you receive enough information?*
   - *Who mainly provided this information?*
   - *Was the care process clear to you at all times?*
9. **Involvement of Family Members:**
   - To what extent were your family members involved in your treatment?
   - *Did they feel included in the process and your care?*
   - *Did they receive sufficient information?*
   - *Were they present during consultations?*
   - *Were they able to support you?*
   - *Which professionals helped involve them?*
10. **Interprofessional Collaboration:**
    - How did you perceive the collaboration between the different professionals? Did you feel they communicated adequately about your current condition, needs, and next steps?
    - *Did you know which professional belonged to which team?*
11. **Point of Contact:**
    - Did you always know whom to contact with questions or uncertainties? How did you find your personal care?
    - *In which situations were you unsure?*
    - *How were your questions and uncertainties eventually resolved?*
    - *Did you have a main point of contact?*
12. **Satisfaction with Care Process/Treatment:**
    - Were you satisfied with the care process and your treatment?
    - *Did the care process contribute to your recovery?*
    - *What were your expectations before the study?*
    - *How did you perceive the study’s impact during your treatment?*
    - *What is your attitude towards the care you received after the study?*
13. **Rehabilitation Experience:**
    - How did you find your rehabilitation stay? **ERAS+:** How did you feel about being directly transferred to rehabilitation?
    - *How did you feel about the timing of the rehabilitation start?*
    - *Did the rehabilitation stay contribute to your recovery? How?*
    - *How fit did you feel upon discharge?*
14. **Suggestions for Improvement:**
    - What should be changed?
15. **Final Thoughts:**
    - What measures or aspects of your treatment personally helped you the most throughout the entire process?

**If there are no further comments, we have now completed the interview, and I will stop the recording.**

**Notes (for example, any noticeable points during the interview):**
